# Supplementary material for: Synthesis of Thermo-Responsive Block-Graft Copolymer Based on PCL and PEG Analogs, and Preparation of Hydrogel via Click Chemistry
Source: Polymers (Basel). 2019 May 1;11(5):765. doi: 10.3390/polym11050765 (PMC6572280; doi:10.3390/polym11050765)

Figure S1 ^{1}HNMR spectra of the triblock copolymer *m*PEG-*b*-PCL-*b*-*m*PEG (a) and triblock- graft copolymer *m*PEG-*b*-[PCL-*g*-(MEO2MA-*co*-OEGMA)]-*b*-*m*PEG (b)

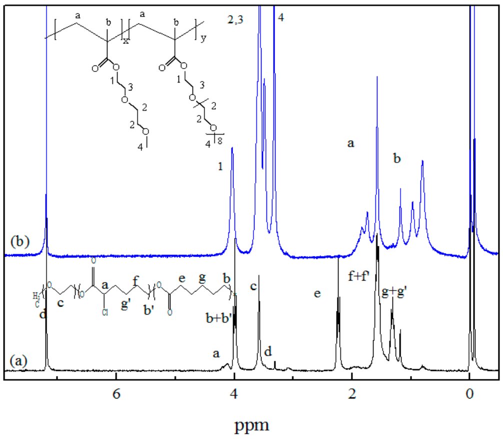

Figure S2 FT-IR spectra of *m*PEG-*b*-[PCL-*g*-(MEO2MA-*co*-OEGMA)]-*b*-*m*PEG triblock-graft copolymer (a) and azide *m*PEG-*b*-[PCL-*g*-(MEO2MA-*co*-OEGMA-*b*-*m*PEG triblock-graft copolymer (b)

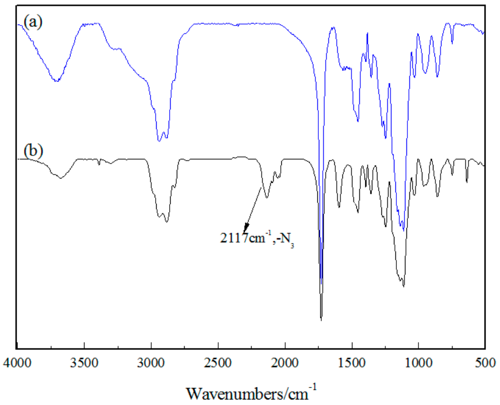

Figure S3 ^{1}HNMR spectra of CTA (a) and P(GMA-*co*-MEO2MA-*co*-OEGMA) (b)

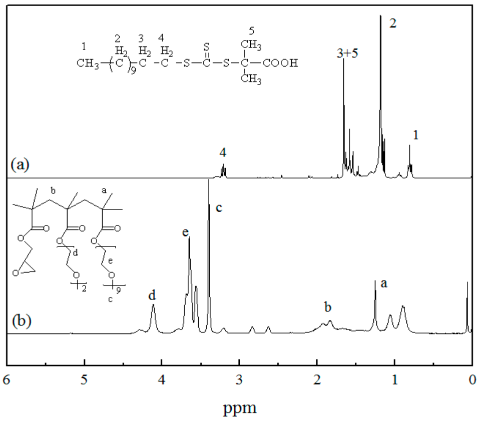

Figure S4 FT-IR spectra of P(GMA-*co*-MEO2MA-*co*-OEGMA) and alkynyl P(GMA-*co*- MEO2MA-*co*-OEGMA)

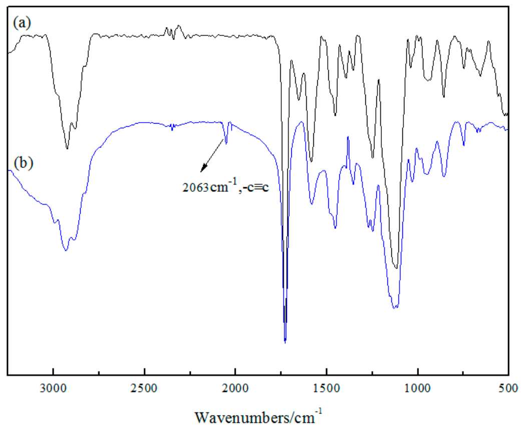

Figure S5 FT-IR spectra of the azide triblock-graft copolymer (a), the crosslinker alkynyl P(GMA-*co*-MEO$_2$MA-*co*-OEGMA) (b), the crosslinker TPOM (c) and the two gels (d, e).

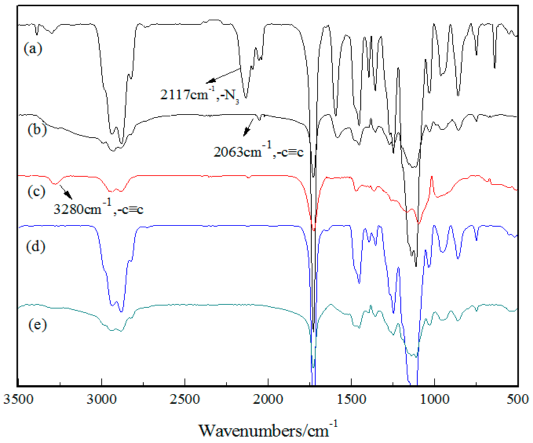

Supplement: Supplementary file 1 [file polymers-11-00765-s001.pdf]
